# Supplementary material for: Photodynamic Effects with 5-Aminolevulinic Acid on Cytokines and Exosomes in Human Peripheral Blood Mononuclear Cells
Source: Biomedicines. 2022 Jan 21;10(2):232. doi: 10.3390/biomedicines10020232 (PMC8869139; doi:10.3390/biomedicines10020232)
Supplement: Supplementary file 1 [file biomedicines-10-00232-s001.zip › biomedicines-1555450-supplementary.pdf]

Supplementary Data

Table S1. Intracellular PpIX in individual subsets of PBMCs after ALA incubation for 4 hours

	PpIX increase (% of controls)				
	**D1**	**D2**	**D3**	**D4**	**Avg.**
**$CD3^+$**	260	292	240	132	231
**$CD4^+$**	270	417	275	113	269
**$CD8^+$**	29	38	26	16	27
**$CD3^-CD19^+$**	536	767	372	243	480
**$CD3^-CD56^+$**	304	150	284	113	213
**$CD11c^+CD14^+$**	327	357	332	55	268

Note: **D1**: donor sample 1; **Avg**: average data of 4 samples from 4 different donors.
The same abbreviations for Tables 2-5.

Table S2. ALA dark cytotoxicity in subsets of PBMCs after ALA incubation for 4 hours

	Cell viability (% of controls without ALA)				
	**D1**	**D2**	**D3**	**D4**	**Avg.**
**$CD3^+$**	90	110	95	95	98
**$CD4^+$**	95	111	99	89	98
**$CD8^+$**	80	119	94	87	95
**$CD3^-CD19^+$**	66	81	83	94	81
**$CD3^-CD56^+$**	12	12	57	45	32
**$CD11c^+CD14^+$**	93	96	61	114	91

Table S3. Killing effects on individual subsets of PBMCs after PDT with ALA

	Cell viability (% of controls with ALA alone)				
	**D1**	**D2**	**D3**	**D4**	**Avg.**
**$CD3^+$**	74	82	42	87	71
**$CD4^+$**	47	28	16	73	41
**$CD8^+$**	74	92	55	83	76
**$CD3^-CD19^+$**	10	11	7	22	13
**$CD3^-CD56^+$**	21	9	1	21	13
**$CD11c^+CD14^+$**	34	20	45	30	32

Table S4. PDT effect on cytokines with ALA

	Log decrease				
	D1	D2	D3	D4	Avg.
**MIP-1alpha**	14,2	17,3	16,0	15,5	16,1
**MIP-1beta**	13,3	13,8	14,1	13,7	13,7
**IL-6**	7,0	13,6	14,4	14,1	13,7
**G-CSF**	10,0	12,0	10,3	10,6	10,9
**IFN-gamma**	3,9	9,5	9,1	9,6	9,0
**IL4**	5,5	8,5	9,6	8,5	8,6
**PDGF-BB**	9,2	7,1	5,7	8,8	8,2
**IL9**	8,0	6,8	9,1	6,1	7,9
**IL-1ra**	0,0	6,6	7,8	7,1	6,8
**IL-5**	5,8	6,0	7,6	6,5	6,7
**IL-15**	4,9	5,3	6,0	4,9	5,4
**IL-12(p70)**	0,0	3,9	4,2	3,9	3,6

Table S5. PDT effect on exosomes with ALA

	Log decrease				
	D1	D2	D3	D4	Avg.
**CD29**	8,6	10,0	9,7	9,1	9,4
**CD9**	7,5	9,0	9,1	9,0	8,8
**CD40**	6,8	8,1	8,9	5,5	7,8
**CD31**	6,5	8,2	8,4	5,3	7,6
**HLA-ABC**	6,5	8,2	8,4	2,4	7,5
**CD41b**	6,3	7,9	8,0	6,9	7,4
**CD69**	6,7	7,3	8,6	3,5	7,4
**HLA-DRDPDQ**	6,1	8,1	7,7	4,3	7,1
**CD44**	4,4	8,0	7,5	6,3	7,1
**CD81**	5,5	7,1	6,9	6,1	6,5
**SSEA-4**	6,2	6,4	7,4	5,0	6,5
**CD8**	5,7	6,9	5,3	4,4	5,9
**CD25**	2,9	5,6	6,4	4,2	5,3
**CD19**	5,0	4,9	5,3	3,9	4,9
**CD4**	3,5	3,5	5,2	4,6	4,4
**CD326**	1,2	2,1	4,2	4,6	3,6
**CD133-1**	4,2	0,0	3,0	2,8	3,1
